# Hypoxic‐hypocapnic red blood cells in PAGGSM additive solution before and after gamma irradiation show improved metabolism

**DOI:** 10.1111/vox.70218

**Published:** 2026-02-23

**Authors:** Soroth Chey, Jacqueline Maier, Samuel Sowemimo‐Coker, Reinhard Henschler

**Affiliations:** ^1^ Institute of Transfusion Medicine, University Hospital Leipzig, Medical Faculty Leipzig University Leipzig Germany; ^2^ Hemanext Inc. Lexington Massachusetts USA

**Keywords:** gamma irradiation, hypoxic‐hypocapnic storage, metabolic changes, red blood cells

## Abstract

**Background and Objectives:**

Hypoxic/hypocapnic (HH) treatment and storage conditions have been shown to reduce oxidative stress and improve red blood cell (RBC) quality. This study aimed to validate a good manufacturing practice HH RBC product for obtaining the licence for routine use in patients, by comparison with normoxic RBCs both without irradiation and after gamma irradiation.

**Materials and Methods:**

Forty‐four units of leucoreduced red cell concentrates (LR‐RCCs) were prepared from 500 mL citrate–phosphate–dextrose (CPD)/phosphate‐adenine‐glucose‐guanosine‐saline‐mannitol (PAGGSM) whole‐blood donations. Paired units of ABO‐matched LR‐RCCs were pooled and split into equal aliquots. Twenty‐two units (control) were maintained at room temperature for 3 h, while 22 units underwent HH treatment for 3 h using the Hemanext ONE® system. Eleven control and 11 HH LR‐RCCs were assigned to the non‐irradiated arm, and the remaining 22 units (11 control, 11 HH) were irradiated on Day 14. In vitro RBC quality was measured until Day 42 (non‐irradiated) and Day 28 (irradiated on Day 14).

**Results:**

The HH product was comparable to controls in haemoglobin (Hgb) content, haematocrit, potassium and haemolysis, both without irradiation over 42 days and after irradiation over 28 days. HH RBCs contained 1.2‐fold higher adenosine triphosphate (ATP) and up to 6.2‐fold higher 2,3‐diphosphoglycerate (2,3‐DPG) levels. Lactate was 25% increased, and glucose levels were up to 30% decreased compared with normoxic RBCs.

**Conclusion:**

HH storage enhanced RBC ATP and 2,3‐DPG levels and yielded a product fulfilling RBC quality criteria for the other parameters. These findings support its clinical use for optimizing RBC transfusion quality, particularly for irradiated units.

**Highlights**

- Red blood cells (RBCs) stored under hypoxic/hypocapnic (HH) conditions exhibited higher adenosine triphosphate (ATP) and 2,3‐diphosphoglycerate (2,3‐DPG) levels compared to normoxic storage, both in non‐irradiated and irradiated arms, suggesting improved metabolic preservation.
- Although haemolysis was slightly higher in irradiated HH RBCs compared to non‐irradiated units, the levels were below 0.8%, meeting the regulatory guideline.
- RBCs stored under HH conditions showed greater glucose consumption and lactate accumulation, reflecting higher glycolytic activity, which may contribute to improved energy homeostasis during storage.

## INTRODUCTION

Red blood cell (RBC) transfusion remains a critical therapy for patients with anaemia or acute blood loss. However, the quality of conventionally stored RBCs deteriorates over time as a result of biochemical and metabolic changes collectively termed ‘storage lesions’, which include adenosine triphosphate (ATP) depletion, loss of 2,3‐diphosphoglycerate (2,3‐DPG), morphological changes, oxidative damage, loss of deformability and haemolysis [1, 2, 3, 4]. These alterations impair RBC functionality and post‐transfusion survival, potentially compromising patient outcomes [5, 6, 7, 8]. For immunocompromised patients, particularly those undergoing haematopoietic stem cell transplantation, gamma irradiation of RBC units is required to prevent transfusion‐associated graft‐versus‐host disease (TA‐GVHD) [9, 10, 11]. While gamma irradiation is primarily recognized for inducing membrane lipid peroxidation and potassium leakage, several studies have also demonstrated its adverse effects on red cell energy metabolism. Specifically, irradiation accelerates ATP depletion and 2,3‐DPG loss by generating reactive oxygen species that inactivate glycolytic enzymes and disrupt membrane transport systems. This metabolic impairment contributes to the overall storage lesion [12, 13, 14]. To address these storage‐related challenges, alternative strategies such as optimized additive solutions, novel storage materials and controlled O_2_/CO_2_ environments have been investigated [15, 16, 17, 18]. Among these, hypoxic/hypocapnic (HH) storage, characterized by reduced oxygen (O_2_) and carbon dioxide (CO_2_) exposure, has gained attention for its potential to mitigate oxidative damage and preserve RBC metabolic integrity [18, 19, 20]. Prior studies have demonstrated that HH storage can maintain higher ATP and 2,3‐DPG levels, reduce oxidative stress, improve RBC post‐transfusion recovery and facilitate faster kinetics of oxygen release compared to conventional normoxic storage [20, 21, 22, 23]. Additionally, recent findings suggest that modifying CO_2_ levels during storage (hypocapnic condition) may further optimize RBC metabolism by preventing intracellular acidification and maintaining the balance between ATP production and consumption [24]. Recent studies have provided comprehensive insights into the effects of irradiation on RBC storage lesions, showing that HH storage conditions may counteract some of these detrimental effects by preserving energy metabolism and reducing oxidative injury [25, 26]. These findings underscore the need for optimized storage strategies to mitigate irradiation‐induced damage. In this study, we compared the effects of normoxic and HH storage conditions on the biochemical and structural integrity of RBCs before and after gamma irradiation. We hypothesized that HH storage will mitigate key markers of the storage lesion and improve RBC quality, even after irradiation. Our primary endpoints include ATP and 2,3‐DPG levels, haemolysis, lactate production and glucose consumption. The findings from this study would support the implementation and regulatory approval of an HH storage strategy for improving the quality of gamma‐irradiated and non‐irradiated RBCs for transfusion.

## MATERIALS AND METHODS

### Blood products and processing

Whole blood (WB; 500 mL) was collected from healthy donors into quadruple bag LQT blood collection systems (LQT742C, MacoPharma, Mouvaux‐France) for the preparation of plasma, buffy coat and leucoreduced red cell concentrate (LR‐RCC). After collection in 70 mL citrate–phosphate–dextrose (CPD), WB units were processed within 8 h of collection, and all LR‐RCCs were placed in cold storage (4°C) within 12 h of venipuncture. The blood was centrifuged at 5000*g* and separated as described previously [27]. The additive solution used for RBC storage was phosphate‐adenine‐glucose‐guanosine‐saline‐mannitol (PAGGSM).

**FIGURE 1:**
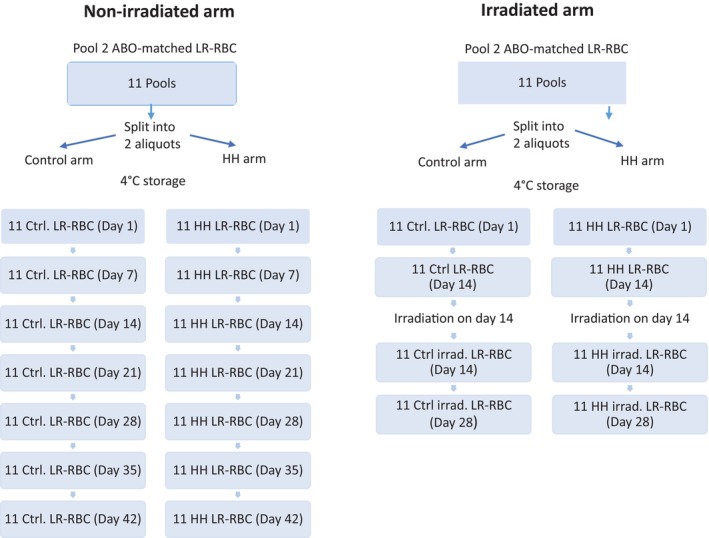
Overview of the experimental design for the comparative validations. Of leucoreduced red blood cell (LR‐RBC) (*n* = 44), two each were ABO‐matched, pooled and then split into conventional storage product (control) and hypoxic/hypocapnic (HH) storage product. gamma irradiation was applied on Day 14.

To ensure the best possible comparability, a matched pair pool‐and‐split experimental design was chosen. For each test, 2 units of ABO‐matched LR‐RCCs were transferred into a 2‐L CompoFlex® transfer bag (R6R2041, Fresenius) under aseptic conditions using a sterile connecting device (Terumo Europe NV, Leuwen, Belgium) and then split into two equal aliquots (300 ± 10 mL each for control [Ctrl] and HH). A total of 44 units of LR‐RCCs were produced. For the non‐irradiated part of the study, 22 units of LR‐RCC were used (11 control and 11 HH). Similarly, 22 units of LR‐RCC were used for the irradiated part of the study (Figure 1). The control units were kept at room temperature for 3 h, while the HH units were oxygen‐reduced using the Hemanext ONE® system according to manufacturer's instructions (Hemanext, Lexington, MA). For HH treatment, the LR‐RCCs bags were sterile‐connected to the Hemanext ONE® oxygen reduction bag set (ORB, Hemanext, Lexington, MA, USA) and RBCs were transferred into the ORB by gravity. The ORB was then placed on a linear shaker and agitated at 72 cycles/min (CPM) for 3 h ± 15 min at 22 ± 2°C for reduction of O_2_ and CO_2_. Following the agitation step, the LR‐RCCs were transferred to the accessory Hemanext ONE® storage bag (HSB).

Both the control and HH units were then stored at 4°C for the maximum storage period of 42 days in the non‐irradiated arm and up to 28 days (14 days after irradiation) in the gamma irradiation arm. Day 14 was selected for irradiation to reflect standard practice, providing a 14‐day post‐irradiation shelf‐life and enabling analysis of storage‐related changes before and after irradiation. gamma Irradiation with a minimum dose of 30 Gy was applied using a RadGil 2 Accelerator (Gamma Service Medical, Leipzig, Germany). Routine dosimetry confirmed a mean central dose of 32 Gy (range 30–34 Gy).

### Sample processing and analyses

At each time point, 25 mL samples were collected using a sterile syringe needle docked to the storage bag with a sterile adapter (Figure 1). Five millilitres was used to quantify haematocrit (HCT), total haemoglobin (Hgb), residual white blood cells and residual platelets using a fluorescence flow cytometer (Sysmex XN 1000, Thermo Fischer, Germany). In parallel, 5 mL of the RBCs was centrifuged at 4000 rpm (2415*g*) for 20 min at room temperature (Universal 320 benchtop centrifuge, Hettich, Tuttlingen, Germany). One millilitre of the supernatant was used for measuring the free Hgb by photometry (Specord 50 Plus, Bioanalytics, Jena, Germany). To test the donor blood for sickle cell anaemia, 100 μL of the LR‐RCC was analysed using the Sickle SCAN® test kit (BioMedomics, Morrisville, USA). Five millilitres was used to measure the potassium, sodium, lactate and pH values on the cobas® b 123 blood gas system (Roche). For determination of ATP and 2,3‐DPG, 3 mL of LR‐RCC was deproteinized with 3 mL ice‐cold 12% (w/v) trichloroacetic acid. After 1 min shaking and 5 min incubation on ice, the tube was centrifuged at 3600*g* for 20 min at 4°C. One‐millilitre aliquots of the protein‐free supernatant were transferred into cryotubes and stored at −80°C. The concentrations of ATP in the samples were measured with the DiaSys ATP Hexokinase Kit for measuring ATP in RBCs (DiaSys Diagnostic System GmbH, Holzheim, Germany), and the concentration of 2,3‐DPG was analysed with an enzymatic assay [28]. For this, intracellular 2,3‐DPG concentrations in RBCs were determined from trichloroacetic acid extracts as described above using a spectrophotometric microtitre plate endpoint assay measured at 340 nm, based on Keith's nicotinamide adenine dinucleotide (NADH)‐linked enzymatic method [28]. In this coupled assay, monophosphoglycerate mutase (PGM; Abcam, Waltham, MA, USA) catalyses the specific phosphatase conversion of 2,3‐DPG to 3‐phosphoglycerate (3‐PGA). The generated 3‐PGA is subsequently processed by phosphoglycerate kinase (PGK; Abcam), which phosphorylates 3‐PGA to 1,3‐diphosphoglycerate using ATP (Sigma‐Aldrich, St. Louis, MO, USA), and by glyceraldehyde‐3‐phosphate dehydrogenase (GAPDH; Abcam), which reduces 1,3‐diphosphoglycerate to glyceraldehyde‐3‐phosphate with stoichiometric oxidation of reduced NADH (Sigma‐Aldrich) to its oxidized form (NAD^+^). The resulting decrease in NADH absorbance at 340 nm is directly proportional to the concentration of 2,3‐DPG in the sample. The percent oxygen saturation of the Hgb of the RBCs (%sO_2_) was determined on the ABL90 Flex blood gas analyser with co‐oximeter (Radiometer GmbH, Krefeld, Germany). Microbiological control was carried out on Day 42 (non‐irradiated arm) and Day 28 (irradiated arm) using the BacT/Alert blood culture system (Bio Merieux, Germany).

### Data analysis and statistics

GraphPad Prism (GraphPad Software, LLC., San Diego, CA, USA) was used for data presentation and statistical analyses. Blood parameter measures were tested by one‐way analysis of variance (ANOVA) with Bonferroni's multiple comparison post‐test. One‐way ANOVA was selected to enable direct pairwise comparisons between the control and HH groups at each specific storage time point, reflecting clinically relevant decision intervals.

## RESULTS

All units met the local specification for volume (200–350 mL) at the start of storage. Despite repetitive sampling, minimum volume thresholds were maintained throughout the storage period, ensuring transfusion suitability at end of shelf‐life (Table S1).

### O_2_ , CO_2_ and pH

**FIGURE 2:**
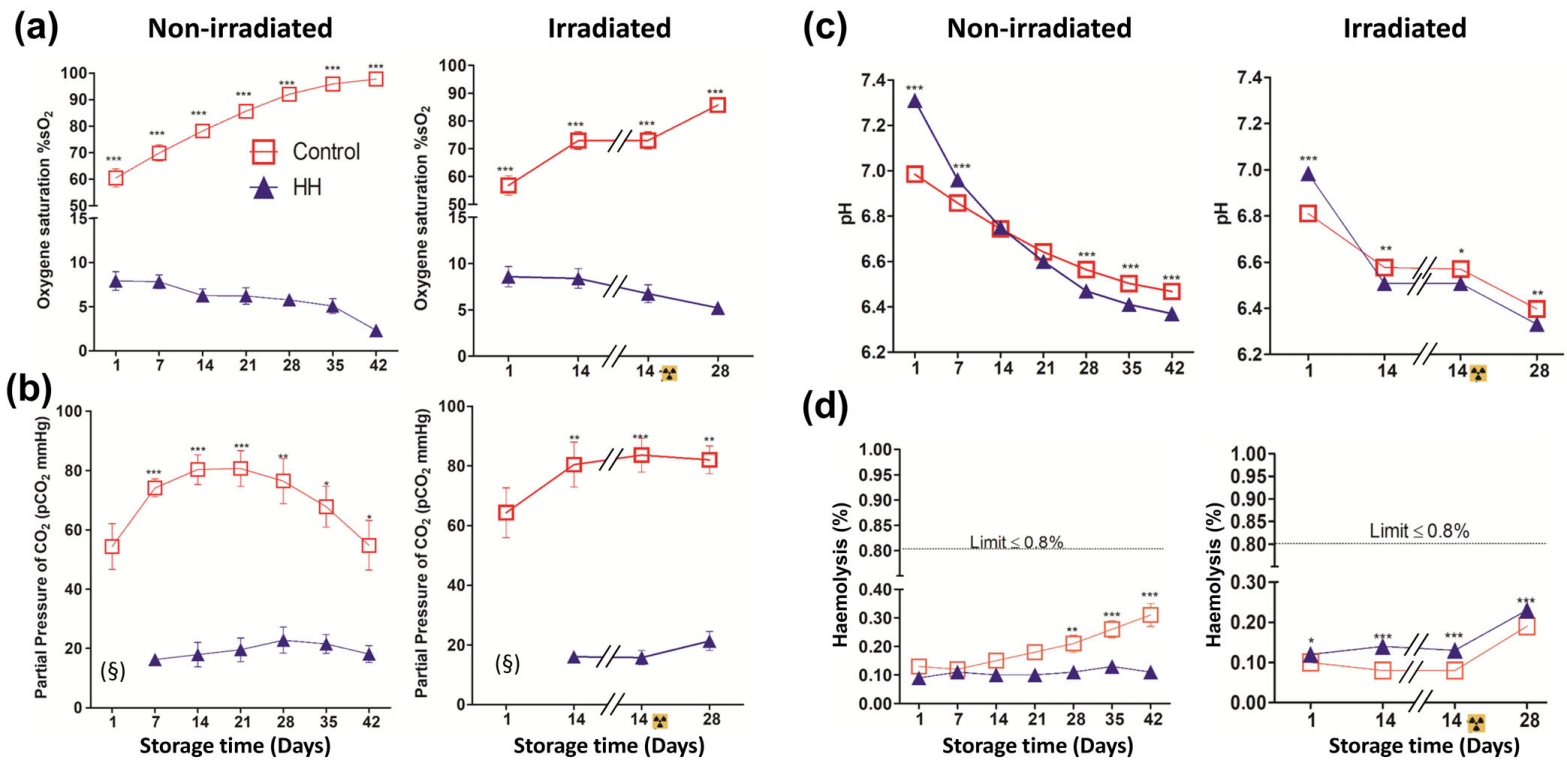
O_2_, CO_2_, pH and haemolysis during hypoxic/hypocapnic (HH) storage versus controls. Samples of red blood cell (RBC) were taken at the indicated time points and analysed as described in Materials and Methods. Open symbols represent control products, and closed symbols represent HH products. Values are means ± SD; *n* = 11 per arm. § indicates concentration was below the detection limit of the method, 12 μM. Statistical significance was calculated using one‐way analysis of variance (ANOVA) test with the additional Bonferroni comparison test of pairs; *p < 0.05; **p < 0.01; ***p < 0.001.

The %sO_2_ in the conventionally stored irradiated and non‐irradiated RBCs increased significantly during storage. However, pCO_2_ increased and then decreased (Figure 2a,b). In contrast, the %sO_2_ and pCO_2_ of HH RBCs in the non‐irradiated and irradiated arms of the study were significantly reduced after HH treatment and remained lower than control through the storage duration (Figure 2a,b). The levels of pCO_2_ in the samples were around 20% or less in the HH‐treated samples. The differences in %sO_2_ and pCO_2_ between the control and HH‐treated RBCs were significant at all the time points, *p* < 0.05 (Figure 2a,b). The pH values in the HH groups on Day 1 were significantly higher than that of the controls for both irradiated and non‐irradiated samples (Figure 2c; *p* <0.05). This initial increase in pH for the HH groups was followed by a decline during storage. Similar decreases in pH values were observed in the control irradiated and non‐irradiated RBCs (Figure 2c).

### Haematological parameters

Hgb and HCT were similar at the end of storage in both arms and fulfilled the European Directorate for Quality Management (EDQM) guideline limits of >40 g/unit and >0.5 [29], respectively, both with and without irradiation (Table S1). The HH units showed a slightly lower Hgb content on Day 42 (mean 7.2% in non‐irradiated units and mean 7.9% in irradiated) compared to their respective normoxic controls (Table S1), in line with their additional passage into deoxygenation and hypoxic storage bags. Haemolysis levels increased during storage in all treatment conditions but remained well below the EDQM guideline maximum of 0.8% (Figure 2d). In the non‐irradiated arm, haemolysis levels in the HH RBCs were significantly lower than control after Day 21, *p* < 0.05 (Figure 2d). In contrast, in the irradiated arm, haemolysis levels were slightly higher in the HH group (Day 1: 0.12% ± 0.006% vs. 0.10% ± 0.006%; Day 28 (14 days post‐irradiation): 0.23% ± 0.010% vs. 0.19% ± 0.007%; *p* <0.05) (Figure 2d).

### Extracellular potassium (K^+^) and sodium (Na^+^) levels

**FIGURE 3:**
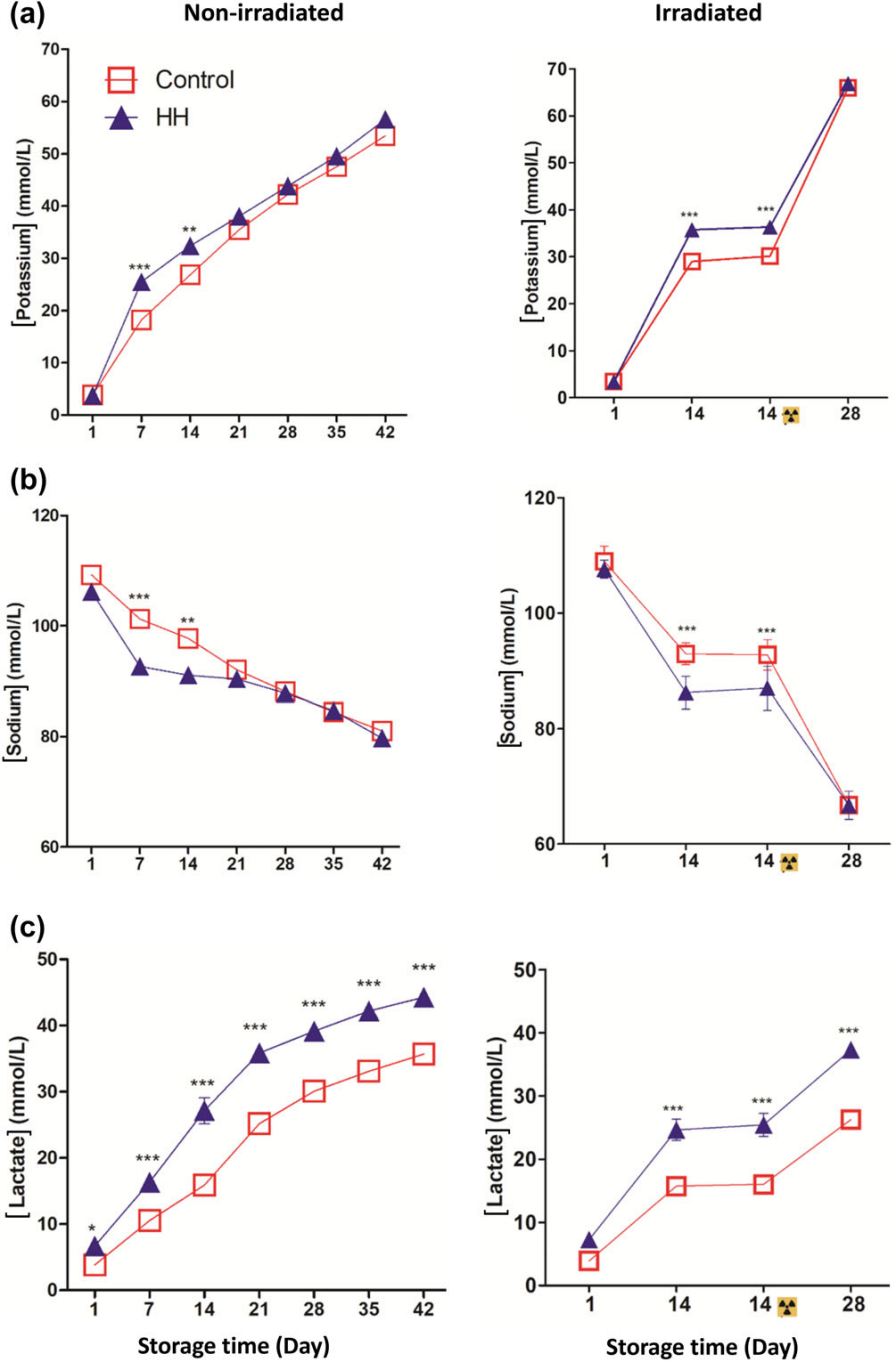
Potassium, sodium, lactate and glucose levels during hypoxic/hypocapnic (HH) storage versus controls. Samples of red blood cell (RBC) were taken at the indicated time points and analysed as described in Materials and Methods. Open symbols represent control products, and closed symbols represent HH products. Values are means ± SD; *n* = 11 per arm. Statistical significance was calculated using one‐way analysis of variance (ANOVA) test with the additional Bonferroni comparison test of pairs; *p < 0.05; **p < 0.01; ***p < 0.001.

The changes in K^+^ and Na^+^ levels in the RBCs as a function of storage duration are shown in Figure 3a,b, respectively. Extracellular K^+^ increased in all treatment conditions as a function of the storage duration (Figure 3a), while the Na^+^ levels decreased during storage (Figure 3b). Potassium levels were very similar, with the exception of the HH arm on days 7 and 14 (Day 7: 25.50 ± 1.44 vs. 18.20 ± 0.69 mmol/L; *p* <0.05), and no significant differences at other intervals. Sodium concentrations were slightly reduced in the HH group on days 7 and 14 but approached control levels thereafter. No differences were observed at Day 28 post irradiation for either electrolyte.

### Lactate accumulation and glucose consumption

**FIGURE 4:**
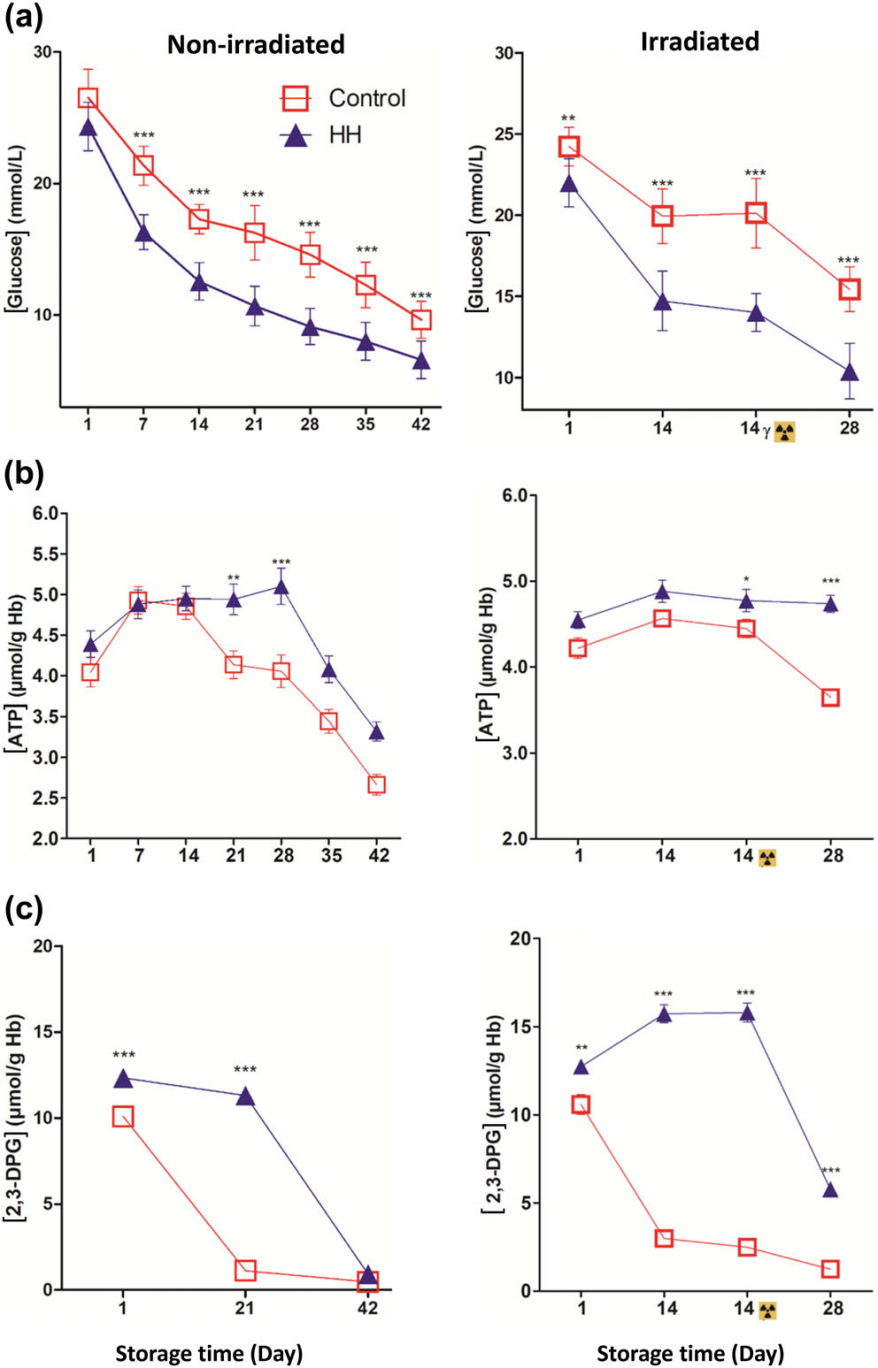
Adenosine triphosphate (ATP) and 2,3‐diphosphoglycerate (2,3‐DPG) levels during hypoxic/hypocapnic (HH) storage versus controls. Samples of red blood cell (RBC) were taken at the indicated time points and analysed as described in Materials and Methods. Open symbols represent control products, and closed symbols represent HH products. Values are means ± SD; *n* = 11 per arm. Statistical significance was calculated using one‐way analysis of variance (ANOVA) test with the additional Bonferroni comparison test of pairs; *p < 0.05; **p < 0.01; ***p < 0.001.

In the non‐irradiated arm, lactate accumulation was consistently higher in the HH group across all time points (Day 1: 6.69 ± 0.16 vs. 3.81 ± 0.10 mmol/L; Day 42: 44.27 ± 0.64 vs. 35.69 ± 0.66 mmol/L; *p* < 0.05). Similarly, in the irradiated arm, lactate accumulation was elevated in the HH group post irradiation (Day 28: 37.31 ± 0.75 vs. 26.25 ± 1.31 mmol/L; *p* < 0.05) (Figure 3c). Conversely, glucose levels were significantly lower in the HH group throughout storage (Day 1: 24.35 ± 0.58 vs. 26.53 ± 0.70 mmol/L; Day 42: 6.59 ± 0.45 vs. 9.64 ± 0.45 mmol/L; *p* < 0.05) and the same trend was noted in the irradiated arm—glucose levels were significantly lower at all time points (Day 1: 22.01 ± 0.45 vs. 24.23 ± 0.36 mmol/L; Day 28 (14 days post irradiation): 10.39 ± 0.52 vs. 15.44 ± 0.41 mmol/L; *p* < 0.05) (Figure 4a). Prior to irradiation (Day 14), lactate concentrations were significantly higher in HH‐stored RBCs compared with normoxic controls (*p* < 0.05), consistent with accelerated glycolytic turnover under low‐oxygen storage conditions. This metabolic shift reflects increased conversion of glucose to lactate as the main pathway for ATP generation in deoxygenated RBCs.

### ATP and 2,3‐DPG production

In the non‐irradiated normoxic RBCs, there was a gradual decrease in the levels of ATP as the storage duration increased from Day 14 to Day 42. In contrast, HH storage prevented the rapid decrease in ATP, and the levels were maintained at higher levels than control throughout the storage duration (Figure 4b). The differences in the ATP levels between normoxic and HH were significant on days 21 and 28 of storage *p* <0.05. Similarly, in the irradiated arm, ATP concentrations were also significantly higher in the HH group post irradiation from Day 1 to Day 28 (Day 14 post irradiation: 4.77 ± 0.13 vs. 4.45 ± 0.11 μmol/g Hgb; Day 28: 4.74 ± 0.10 vs. 3.65 ± 0.08 μmol/g Hgb; *p* < 0.05). Because of a world‐wide shortage of reagents for the 2,3‐DPG assay, the levels of 2,3‐DPG in the RBCs could only be determined only on days 1, 21 and 42 of storage. In the non‐irradiated arm, 2,3‐DPG was significantly higher in HH storage than control on days 1 and 21 (Day 21: 11.31 ± 0.48 vs. 1.12 ± 0.22 μmol/g Hgb; *p* < 0.05) (Figure 4c). Similarly, in the irradiated arm, 2,3‐DPG levels remained significantly higher in the HH group than control from Day 1 to Day 28 (14 days post irradiation) (Figure 4c).

## DISCUSSION

In this study, we evaluated the effects of gamma irradiation on HH RBCs and compared their quality with that of gamma‐irradiated and non‐irradiated normoxic RBCs during refrigerated storage in a good manufacturing practice (GMP) manufacturing setting. The aim of the study was to validate HH RBC in order to apply for a manufacturing licence and marketing authorization. To this end, demonstration that the product was as good as the standard and an irradiated red cell product was the primary goal. Further endpoints included determination of pH, ATP and 2,3‐DPG.

Our study demonstrates that in HH RBCs, Hgb content, HCT, residual leukocytes and residual platelets all fulfil guideline requirements (Table S1). HH RBCs exhibit improved biochemical and metabolic quality compared to conventionally stored normoxic RBCs. HH RBCs maintained significantly higher levels of ATP and 2,3‐DPG. These findings are consistent with previous research suggesting that HH storage mitigates oxidative stress and enhances energy metabolism, thereby preserving RBC functionality [18, 20, 21, 25, 26]. The preservation of ATP and 2,3‐DPG is particularly noteworthy, as these metabolites are crucial for RBC energy metabolism and oxygen delivery capacity, respectively [20, 23]. Additionally, HH RBCs demonstrated lower haemolysis rates than normoxic RBCs, suggesting that the HH storage conditions confer a protective effect against membrane degradation.

Metabolically, HH RBCs displayed higher lactate accumulation and increased glucose consumption compared to normoxic RBCs, indicating enhanced glycolytic activity. This aligns with previous reports suggesting that low‐oxygen environments promote glycolysis, thereby sustaining ATP levels and delaying metabolic deterioration [20, 21]. Although HH RBCs initially exhibited a higher pH compared to normoxic RBCs, the pH declined more rapidly over time, ultimately resulting in a lower pH than normoxic RBCs by Day 42. The accelerated acidification in HH RBCs may be attributed to the increased glycolytic flux and subsequent accumulation of acidic metabolic by‐products, such as lactate and protons. The higher lactate concentrations observed in HH RBCs prior to irradiation indicate increased glycolytic flux rather than metabolic impairment. In deoxygenated RBCs, there is increased glycolysis to maintain ATP production, leading to accumulation of lactate as the terminal glycolytic product. This elevation, therefore, represents a marker of active metabolism rather than deterioration. Although lactate levels were elevated in HH RBCs due to increased glycolysis, the clinical significance of this observation may be negligible since transfused RBCs rapidly equilibrate with recipient plasma and lactate is cleared through metabolic pathways in the liver and other tissues even in patients with lactic acidosis [30, 31].

In mature erythrocytes, which lack mitochondria, enhanced glycolysis under low‐oxygen conditions arises through red cell–specific mechanisms. Deoxygenated Hgb binds to the cytoplasmic domain of band 3 and displaces several glycolytic enzymes from the membrane, increasing their availability and catalytic activity in the cytosol. This displacement enhances glycolytic flux and stimulates the Rapoport–Luebering shunt, leading to higher production of ATP and 2,3‐DPG. In addition, reduced oxygen tension limits oxidative damage to redox‐sensitive enzymes, thereby maintaining glycolytic efficiency throughout storage [20].

Gamma irradiation is known to induce oxidative stress and cellular damage in stored RBCs, leading to increased haemolysis and metabolic degradation [12, 13, 14]. In the irradiated arm of our study, both HH and normoxic RBCs were subjected to gamma irradiation on Day 14 and subsequently stored for an additional 14 days. HH RBCs retained significantly higher ATP and 2,3‐DPG levels than irradiated normoxic RBCs. This suggests that pre‐storage HH conditioning confers some resistance to the metabolic depletion typically induced by irradiation, possibly by reducing oxidative stress and preserving glycolytic enzyme activity. This indicates that HH RBCs may be the superior product of choice if delivering irradiated RBCs.

Our results showed increases in extracellular K^+^ and free Hgb after gamma irradiation in both normoxic and HH RBCs, in agreement with previous studies with normoxic RBCs [25, 26]. A slight increase of K^+^ was found on days 7 and 14, which disappeared at later time points. It is unclear if this reflects the HH treatment or the transfer into two additional bags, which is implied in the HH process. Overall, the guideline requirements were met. While the transient increase in extracellular K^+^ on days 7–14 remained within guideline limits, it may be clinically relevant for large‐volume neonatal transfusions where hyperkalemia poses a risk. Using very fresh (<7 days) or washed HH RBCs may be advisable in such settings. Our study did not assess K^+^ levels immediately after early irradiation, which represents a limitation warranting future investigation.

Also, haemolysis levels in gamma‐irradiated HH RBCs remained well below the EDQM guideline of <0.8% [29], although they were slightly higher in HH RBCs at all storage intervals than control. Interestingly, a previous publication using the same additive solution (PAGGSM) showed lower haemolysis values in gamma‐irradiated HH RBCs compared with normoxic RBCs, but with some haemolysis data well above 0.8 % in both conditions [26]. The slightly higher haemolysis in gamma‐irradiated HH RBCs compared with controls—not seen in Bardyn et al. [26]—may reflect procedural differences. The HH process involves additional bag transfers, possibly rendering RBC membranes marginally more sensitive to irradiation‐induced oxidative stress. The pre‐irradiation difference in haemolysis between arms likely reflects normal biological and procedural variation between independent pooled unit sets, or may be related to differences in preparative procedures and in the initial handling of WB before RBC processing. In our study, the products were processed according to the manufacturer's validated and conformity with European health, safety and envirnonmental protection standards‐marked instructions for use, ensuring compliance with European regulatory standards. Also, in the previous study, WB was stored overnight before the HH procedure [26].

Similar to the non‐irradiated RBCs, the pH of irradiated HH RBCs was initially higher than in normoxic RBCs. This transient alkalinization has been previously reported in RBC storage studies involving HH conditions, where a reduction in CO_2_ minimizes intracellular acidification and may help stabilize RBC metabolism in early storage [24]. In both HH and controls, pH showed a decline over time with values at or above 6.4, at which the functionalities of the RBCs are known to be well maintained [32]. The initial increase in pH observed in HH RBCs can be attributed to the simultaneous induction of hypocapnia during treatment. The removal of CO_2_ reduces the formation of carbonic acid, thereby shifting the bicarbonate equilibrium towards a more alkaline state. However, as storage progresses, glycolytic activity and lactate accumulation contribute to the normal gradual decline in pH over time in both HH and conventionally stored RBCs.

Taken together, these findings indicate the feasibility of the HH process under GMP conditions with a product fulfilling the EDQM guide quality criteria including the total Hgb in 1 unit of RBC for transfusion [29]. The higher ATP and 2,3‐DPG levels in HH RBCs may be particularly beneficial for patients requiring high oxygen availability, such as in critical anaemia, sickle cell disease, in utero transfusions or patients undergoing massive transfusions. The observed lower haemolysis rates in non‐irradiated HH RBCs suggest a reduced risk of haemolysis‐related complications [32].

In conclusion, HH processing of RBCs represents a transformative approach to RBC storage, offering preserved or enhanced product quality both in radiated and non‐irradiated RBCs. The safety of the RBCs is ensured because the RBCs do not come into contact with the oxygen removal material and gas diffusion occurs through the bag membranes. These data provide the basis for accelerated clinical adoption and studying outcomes.

## CONFLICT OF INTEREST STATEMENT

S.S.‐C. is an employee of Hemanext Inc. S.C., J.M. and R.H. declare no conflicts of interest.

## Supporting information


**Table S1** Supporting information.

## Data Availability

The data that support the findings of this study are available from the corresponding author upon reasonable request.

## References

[1] Hess JR . Red cell changes during storage. Transfus Apher Sci. 2010;43:51–59. 20558107 10.1016/j.transci.2010.05.009

[2] Orlov D , Karkouti K . The pathophysiology and consequences of red blood cell storage. Anaesthesia. 2015;70:29–37. 25440392 10.1111/anae.12891

[3] Yoshida T , Prudent M , D'Alessandro A . Red blood cell storage lesion: causes and potential clinical consequences. Blood Transfus. 2019;27:27–52. 10.2450/2019.0217-18 PMC6343598 30653459

[4] Zimring JC . Established and theoretical factors to consider in assessing the red cell storage lesion. Blood. 2015;125:2185–2189. 25651844 10.1182/blood-2014-11-567750 PMC4383795

[5] Triulzi DJ , Year M . Clinical studies of the effects of blood storage on patient outcomes. Transfus Apher Sci. 2010;43:95–106. 20656558 10.1016/j.transci.2010.05.013

[6] Voorhuis FT , Dieleman JM , de Vooght K MK , van Dijk D , van Herwerden LA , Peelen LM , et al. Storage time of red blood cell concentrates and adverse outcomes after cardiac surgery: a cohort study. Ann Hematol. 2013;92:1701–1706. 23832235 10.1007/s00277-013-1832-z

[7] Spinella PC , Carroll CL , Staff I , Gross R , McQuay J , Keibel L , et al. Duration of red blood cell storage is associated with increased incidence of deep vein thrombosis and in hospital mortality in patients with traumatic injuries. Crit Care. 2009;13:R151. 19772604 10.1186/cc8050 PMC2784373

[8] Koch CG , Li L , Sessler DI , Figueroa P , Hoeltge GA , Mihaljevic T , et al. Duration of red‐cell storage and complications after cardiac surgery. N Engl J Med. 2008;358:1229–1239. 18354101 10.1056/NEJMoa070403

[9] Treleaven J , Gennery A , Marsh J , Norfolk D , Page L , Parker A , et al. Guidelines on the use of irradiated blood components. Br J Haematol. 2011;152:35–51. 21083660 10.1111/j.1365-2141.2010.08444.x

[10] Luban NL . Prevention of transfusion‐associated graft‐versushost‐ disease by inactivation of T cells in platelets. Semin Hematol. 2001;38:34–45. 11727284 10.1016/s0037-1963(01)90122-2

[11] Moroff G , Luban NL . The irradiation of blood and blood components to prevent graft‐versus‐host disease: technical issues and guidelines. Transfus Med Rev. 1997;11:15–26. 9031487 10.1016/s0887-7963(97)80006-5

[12] Davey RJ , McCoy NC , Yu M , Sullivan JA , Spiegel DM , Leitman SF . The effect of prestorage irradiation on posttransfusion red cell survival. Transfusion. 1992;32:525–528. 1502705 10.1046/j.1537-2995.1992.32692367195.x

[13] Anand AJ , Dzik WH , Imam A , Sadrzadeh SMH . Radiation induced red cell damage: role of reactive oxygen species. Transfusion. 1997;37:160–165. 9051090 10.1046/j.1537-2995.1997.37297203518.x

[14] Hauck B , Oremek D , Zimmermann R , Ruppel R , Troester B , Eckstein R . Influence of irradiation on in vitro red‐blood cell (RBC) storage variables of leucoreduced RBCs in additive solution PAGGS‐M. Vox Sang. 2011;101:21–27. 21155835 10.1111/j.1423-0410.2010.01455.x

[15] Hess A . An update on solutions for red cell storage. Vox Sang. 2006;91:13–19. 16756596 10.1111/j.1423-0410.2006.00778.x

[16] Hogman CF , de Verdier CH , Ericson A , Hedlund K , Sandhagen B . Effects of oxygen on red cells during liquid storage at 4 degrees C. Vox Sang. 1986;51:27–34. 3090783 10.1111/j.1423-0410.1986.tb00204.x

[17] Lagerberg JW , Korsten H , van der Meer F , de Korte D . Prevention of red cell storage lesion: a comparison of five different additive solutions. Blood Transfus. 2017;15:456–462. 28488968 10.2450/2017.0371-16 PMC5589708

[18] Yoshida T , Shevkoplyas SS . Anaerobic storage of red blood cells. Blood Transfus. 2010;8:220–236. 20967163 10.2450/2010.0022-10 PMC2957487

[19] Meng Q , Peng X , Zhao S , Xu T , Wang S , Liu Q , et al. Hypoxic storage of erythrocytes slows down storage lesions and prolongs shelf‐life. J Cell Physiol. 2019;234:22833–22844. 31264213 10.1002/jcp.28847

[20] D'Alessandro A , Yoshida T , Nestheide S , Nemkov T , Stocker S , Stefanoni D , et al. Hypoxic storage of red blood cells improves metabolism and post‐transfusion recovery. Transfusion. 2020;60:786–798. 32104927 10.1111/trf.15730 PMC7899235

[21] Yoshida T , AuBuchon JP , Tryzelaar L , Foster KY , Bitensky MW . Extended storage of red blood cells under anaerobic conditions. Vox Sang. 2007;92:22–31. 17181587 10.1111/j.1423-0410.2006.00860.x

[22] Bencheikh L , Nguyen KA , Chadebech P , Kiger L , Bodivit G , Jouard A , et al. Preclinical evaluation of the preservation of red blood cell concentrates by hypoxic storage technology for transfusion in sickle cell disease. Haematologica. 2022;107:1944–1949. 35354249 10.3324/haematol.2021.279721 PMC9335112

[23] Rabcuka J , Blonski S , Meli A , Sowemimo‐Coker S , Zaremba D , Stephenson D , et al. Metabolic reprogramming under hypoxic storage preserves faster oxygen unloading from stored red blood cells. Blood Adv. 2022;6:5415–5428. 35736672 10.1182/bloodadvances.2022007774 PMC9631703

[24] Dumont LJ , D'Alessandro A , Szczepiorkowski ZM , Yoshida T . CO_2_ dependent metabolic modulation in red blood cells stored under anaerobic condition. Transfusion. 2016;56:392–403. 26477888 10.1111/trf.13364 PMC4752401

[25] Sowemimo‐Coker SO , Fast LD . Effects of hypoxic storage on the efficacy of gamma irradiation in abrogating lymphocyte proliferation and on the quality of gamma‐irradiated red blood cells in additive solution 3. Transfusion. 2021;61:3443–3454. 34671985 10.1111/trf.16683

[26] Bardyn M , Crettaz D , Borlet M , Langst E , Martin A , Abonnenc M , et al. Hypoxia and hypocapnia storage of γ‐irradiated red cell concentrates. Blood Transfus. 2021;19:300–308. 32955427 10.2450/2020.0075-20 PMC8297680

[27] Hoppe F , Maier J , Kirsten H , Federbusch M , Henschler R . Split red blood cell units contain defined extracellular K^+^ levels, which are improved by a washing procedure. Vox Sang. 2025;120:1066–1073. 39962346 10.1111/vox.70004 PMC12602131

[28] Keitt AS . Reduced nicotinamide adenine dinucleotide‐linked analysis of 2,3‐diphosphoglyceric acid: spectrophotometric and fluorometric procedures. J Lab Clin Med. 1971;77:470–475. 4324263

[29] Council of Europe . Guide to the preparation, use and quality assurance of blood components. 20th ed. Strasbourg, France: Council of Europe Publishing; 2020.

[30] Dhabangi A , Ainomugisha B , Cserti‐Gazdewich C , Ddungu H , Kyeyune D , Musisi E , et al. Effect of transfusion of red blood cells with longer vs shorter storage duration on elevated blood lactate levels in children with severe anemia. The TOTAL randomized clinical trial. JAMA. 2015;314:2514–2523. 26637812 10.1001/jama.2015.13977

[31] Czempik PF , Gierczak D , Wilczek D , Krzych LJ . The impact of red blood cell transfusion on blood lactate in non‐bleeding critically ill patients. A retrospective cohort study. J Clin Med. 2022;11:1037. 35207310 10.3390/jcm11041037 PMC8879325

[32] Hess JR . Measures of stored red blood cell quality. Vox Sang. 2014;107:1–9. 10.1111/vox.12130 24446817

